# Phospholipid scramblase 1 amplifies anaphylactic reactions *in vivo*

**DOI:** 10.1371/journal.pone.0173815

**Published:** 2017-03-10

**Authors:** Asma Kassas-Guediri, Julie Coudrat, Emeline Pacreau, Pierre Launay, Renato C. Monteiro, Ulrich Blank, Nicolas Charles, Marc Benhamou

**Affiliations:** 1 INSERM U1149, Faculté de Médecine Xavier Bichat, Paris, France; 2 University Paris-Diderot, Sorbonne Paris Cité, Laboratoire d’excellence INFLAMEX, DHU FIRE, Paris, France; Centre National de la Recherche Scientifique, FRANCE

## Abstract

Mast cells are critical actors of hypersensitivity type I (allergic) reactions by the release of vasoactive and proinflammatory mediators following their activation by aggregation of the high-affinity receptor for immunoglobulin E (FcεRI). We have previously identified Phospholipid Scramblase 1 (PLSCR1) as a new molecular intermediate of FcεRI signaling that amplifies degranulation of the rat mast cell line RBL-2H3. Here we characterized primary mast cells from *Plscr1*^*-/-*^ mice. The absence of PLSCR1 expression did not impact mast cell differentiation as evidenced by unaltered FcεRI expression, general morphology, amount of histamine stored and expression of FcεRI signal effector molecules. No detectable mast cell deficiency was observed in *Plscr1*^*-/-*^ adult mice. In dose-response and time-course experiments, primary cultures of mast cells (bone marrow-derived mast cells and peritoneal cell-derived mast cells) generated from *Plscr1*^*-/-*^ mice exhibited a reduced release of β-hexosaminidase upon FcεRI engagement as compared to their wild-type counterparts. *In vivo*, *Plscr1*^*-/-*^ mice were protected in a model of passive systemic anaphylaxis when compared to wild-type mice, which was consistent with an observed decrease in the amounts of histamine released in the serum of *Plscr1*^*-/-*^ mice during the reaction. Therefore, PLSCR1 aggravates anaphylactic reactions by increasing FcεRI-dependent mast cell degranulation. PLSCR1 could be a new therapeutic target in allergy.

## Introduction

Mast cells are involved in immune surveillance, inflammatory reactions and antibacterial/antiparasitic defenses [1, 2]. They are also main actors of hypersensitivity type I (allergic) reactions by the release of proinflammatory (preformed and newly synthesized) mediators following their activation through the high-affinity receptor for immunoglobulin E (FcεRI) [1]. FcεRI signaling is composed of multiple parallel, sequential and interconnected pathways such as the ones initiated by the Src-family tyrosine kinases Lyn and Fyn [3, 4]. These pathways involve the activation of the tyrosine kinase Syk, the phosphorylation of multiple signal intermediates such as the adaptors LAT1 and LAT2 and the mobilization of calcium. They result in the release of mast cell granule content into the extracellular milieu, in the production of arachidonic acid metabolites and in the secretion of various cytokines and growth factors [5]. How this complex signaling network is regulated is still a challenging open question for ongoing research programs.

We have previously identified the Phospholipid scramblase 1 (PLSCR1) as a regulator in FcεRI signaling [6]. PLSCR1, as its name suggests, was originally identified for its membrane phospholipid scrambling ability as demonstrated by *in vitro* experiments with reconstituted proteoliposomes [7]. However, to this day, its physiological role in the disruption of the asymmetric distribution of phospholipids in the plasma membrane was not confirmed *in vivo* [8]. Recently, other proteins with a phospholipid scramblase activity have been identified (anoctamins, Xkr8, rhodopsin) [9–11] and PLSCR1 appears to fulfill many other functions. These include regulation of cell proliferation, differentiation, apoptosis and tumor development [3, 12–20], regulation of antiviral immunity [21–26] and of signaling by receptors to many growth factors (EGF, SCF and G-CSF) [8, 27] and by FcεRI [6].

We reported that PLSCR1 is highly phosphorylated on tyrosine residues following the engagement of FcεRI in the RBL-2H3 rat mast cell line [28] and in mouse bone marrow-derived cultured mast cells (BMMC) [29]. We also reported recently that tyrosine phosphorylation of PLSCR1 is subject to a complex regulation downstream of FcεRI aggregation [29]. Thus, it relies on Lyn and Syk but depends only partially on calcium mobilization while Fyn negatively regulates it. This multiplicity of regulatory mechanisms suggested that PLSCR1 might play important roles in FcεRI-dependent mast cell activation. Indeed, using an shRNA approach to repress its expression in the RBL-2H3 rat mast cell line, we observed that PLSCR1 amplifies degranulation and VEGF production without any effect on the production of leukotrienes, prostaglandins and MCP-1 [6].

These results were obtained in a tumoral mast cell line. The present study was conducted to validate the role of PLSCR1 in non-tumoral mast cells and to further explore it *in vivo*. We report that PLSCR1 amplifies anaphylactic reactions *in vivo* through amplification of IgE/antigen-induced mast cell degranulation.

## Materials and methods

### Ethics statement

Mice were maintained and used in accordance with INSERM guidelines and Animal Study Proposal (n°5283) approved by the French ministry for higher education and research. All injections were made under Vetflurane anesthesia and all efforts were made to minimize suffering of the animals. No animal died during the *in vivo* experiments and animal conditions were checked first daily, then every five minutes during the course of these experiments until euthanasia. Euthanasia were made by CO2 asphyxia.

### Mice

Mice invalidated for the *Plscr1* gene were previously described [8]. These mice were obtained from the European Mouse Mutant Archives under a mixed C57BL6/129Sv background. Consequently, we backcrossed them one time in C57BL6 background and used mice of the same sibship as *Plscr1*^*-/-*^ and WT controls for *in vivo* and *in vitro* studies.

### Antibodies

The anti-mouse PLSCR1 monoclonal antibody 1A8 has been described elsewhere [27] and was a generous gift of Dr. P.J. Sims (University of Rochester, Rochester, NY). The anti-Syk polyclonal antibody has been described [30]. Anti-Lyn, anti-Fyn, anti-LAT and anti-ERK antibodies were from Santa-Cruz Biotech (Santa-Cruz, CA). Anti-Akt and anti-PLCγ1 were from Cell Signaling Technology (Danvers, MA). Anti-DNP mouse monoclonal IgE clone DNP48 [31] was a kind gift of Dr. R.P. Siraganian (NIDCR, NIH, Bethesda, MD). Anti-actin and horseradish peroxidase-labeled secondary antibodies were from Sigma-Aldrich (St Louis, MO).

### Generation and culture of mast cells

To generate BMMC, bone marrow cells from WT or *Plscr1*^*-/-*^ mice were cultured in IMDM-Glutamax medium containing 15% fetal calf serum, 25 mM HEPES, 1 mM sodium pyruvate, 1% non-essential amino acids (GIBCO® by Life technologies), 100 U/ml penicillin and 100 μg/ml streptomycin (Life technologies), supplemented with 10 ng/ml interleukin-3, with or without 10 ng/ml Stem Cell Factor (SCF). From the third week on, cells are sown at 1x10^6^/ml at each change of medium. Cells were fully differentiated into mast cells (as evidenced by flow cytometry analysis) and in sufficient numbers between the 4th and 6th week of culture. To generate PCMC, cells from peritoneal lavage of WT or *Plscr1*^*-/-*^ mice were grown in the same conditions as BMMC. Mast cells of both origins were used for the experiments between 4 and 9 weeks of culture.

### Mast cell stimulation

Mast cells (BMMC or PCMC) at 1x10^6^/ml were plated overnight with 1:250 dilution of ascitic fluid containing anti-DNP IgE clone DNP48. Cells were washed two times in Tyrode's solution (NaCl 135 mM, KCl 5 mM, glucose 5.6 mM, CaCl2 1.8 mM, MgCl2 1 mM, BSA 1 mg/ml, HEPES 10 mM pH 7.4). Mast cells were stimulated with the antigen DNP-HSA at the optimal concentration of 10 ng/ml for different times or at different concentrations of antigen for 30 minutes at 37°C. The stimulation was stopped by cooling of the cell suspension in a mixture of water and ice. Following a centrifugation at 450g for 5 min at 4°C, the supernatant was recovered to quantify the extent of degranulation.

### Degranulation measurements

Degranulation was assessed by measurement of the release of the granule marker β-hexosaminidase as described [32]. Briefly, the total amount of this enzyme contained in cells was evaluated after lysis of unstimulated cells with 0.5% Triton-X100. In a 96-well plate, 5μl of unstimulated or stimulated cell supernatant or of cell lysate and 45 μl of β-hexosaminidase substrate solution containing para-nitrophenyl-N-acetyl-β-D-glucosaminide (Sigma), were mixed and incubated for 2 hours at 37°C. The reaction was stopped by addition of 150 μl of 0.2M glycine, pH 10.7. The optical density was measured at a wavelength of 405 nm with a plate reader (Infinite M200, Tecan). The percent of β-hexosaminidase released was then calculated relative to its total amount in non-stimulated cells.

### Cell lysis and immunoblotting

The pellet of 1x10^6^ cells stimulated or not was lysed with 200μl of lysis buffer (50 mM Tris-HCl pH 7.2, 100 mM NaCl, 50 mM NaF, 1 mM Na3VO4, 1% Triton X100, protease and phosphatase Inhibitor Cocktail EDTA-free 1X (Thermo Scientific)). After 10 min on ice, the soluble cell lysates were recovered following a centrifugation at 14,000 g for 10 min at 4°C, then boiled for 5 minutes in Laemmli sample buffer.

Proteins were resolved by SDS-PAGE (10%), transferred onto PVDF membranes and immunoblotting was performed. Membranes were saturated by a 1-hour incubation in TTBS (Tris-HCl 10 mM pH 7.4, 150 mM NaCl, 0.05% Tween-20) containing 4% BSA, then incubated with the desired primary antibody diluted in TTBS-4% BSA for 1hr, washed 3 times with TTBS for 10 min each and incubated with the relevant secondary antibody (anti-mouse or anti-rabbit IgG) coupled with horseradish peroxidase (HRP) (GE healthcare) (1:40,000 dilution) in TTBS 4% BSA followed by 3 washes with TTBS for 10 min. Blots were revealed by chemiluminescence using the kit Super Signal West Pico Chemiluminescent Substrate (Thermo Scientific®) and exposure to photographic film (Kodak). Loading controls were obtained after stripping of the membranes of the first round antibodies and blotting with anti-actin antibodies.

### Analysis of mast cells by flow cytometry

Purity of the mast cell cultures was confirmed by double positivity for anti-CD117 and anti-FcεRI antibody labeling. Cells were washed twice in PBA (PBS containing 1% BSA and 0.05% sodium azide) and incubated for 15 min at 4°C with 60 μl ascitic fluid containing 2.4G2 monoclonal antibody to block IgG receptors. Cells were then incubated for 30 min at 4°C with AF647-conjugated anti-mouse FcεRIα chain (clone MAR1), APC/Cy7-conjugated anti-mouse CD117 (clone 2B8) or an isotype control (all from BioLegend, San Diego CA). After two washes in PBA, cells were resuspended in 200 μl PBA and analyzed using a flow cytometer FACSCantoII.

### Staining of mast cells

For cultured mast cells, approximately 150,000 cells were centrifuged for 2 min at 600 rpm using a cytospin centrifuge, allowing their adhesion on a glass slide. Cells were stained according to two methods: 1-Staining with May-Grünwald-Giemsa (MGG) with the Accustain Sigma kit according to the manufacturer’s protocol. 2-Staining with toluidine blue (TB): Slides were stained with TB (0.2 g in a solution of PBS containing 50% ethanol and adjusted to pH 1) for 30 min and then gently rinsed with water. Slides were dried, mounted with the Eukitt mounting medium and observed under an optical microscope.

For staining of tissues, sections of tissues embedded in paraffin were incubated with toluidine blue for 5 to 10 minutes, rinsed, dried and mounted with Eukitt medium. The same tissues from W^sh^ mice (which are mast cell-deficient) were used as negative controls for the staining.

### Passive systemic anaphylaxis (PSA)

Passive systemic anaphylaxis (PSA) was carried out on mice aged 10 to 12 weeks. Mice were injected intravenously (i.v.) with anti-DNP IgE monoclonal antibody DNP48 (20 μg/mouse) and a thermal probe (model IPTT-300, PLEXX, The Netherlands) was placed under the dorsal skin of the mice mice under Vetflurane anesthesia. Twenty-four hours later they were injected i.v. with DNP-HSA antigen at an optimal dose (2 μg/g of mouse). PSA was monitored by measurement of the drop in body temperature after antigen injection using a reader for thermal probes (PLEXX, Elst, Netherlands). For some mice, blood was drawn 25 minutes after injection of antigen to quantify the histamine released in the serum. In some cases, thermal probes were inserted under the dorsal skin of wild-type (WT) and *Plscr1*^*-/-*^ (KO). The following day mice were injected i.v. with 5 μmol histamine and the drop in their body temperature was monitored.

All injections were made under Vetflurane anesthesia and all efforts were made to minimize suffering of the animals. No animal died during the *in vivo* experiments and animal conditions were checked first daily, then every five minutes during the course of these experiments. Euthanasia were made by CO2 asphyxia.

### Histamine measurement

Mast cells collected from peritoneal cavity lavage were counted in an aliquot of the lavage after their staining with toluidine blue. In the other aliquot cells were lysed in water by osmotic shock and histamine was measured with an EIA kit (Bertin Pharma, Montigny-le-Bretonneux, France) following the instructions of the manufacturer. The amount of histamine stored per peritoneal mast cell was then determined after calculating the ratio between the total amount of histamine and the number of mast cells.

The concentration of histamine released in mouse serum 25 min after induction of PSA was determined using the same EIA kit.

### Statistical analyses

All experiments were conducted at least three times (see figure legends). Statistical analyses were performed using GraphPad Prism 5.0 as indicated in figure legends.

## Results

### The knocking-out of PLSCR1 expression does not impact mast cell differentiation *in vitro*

**Fig 1:**
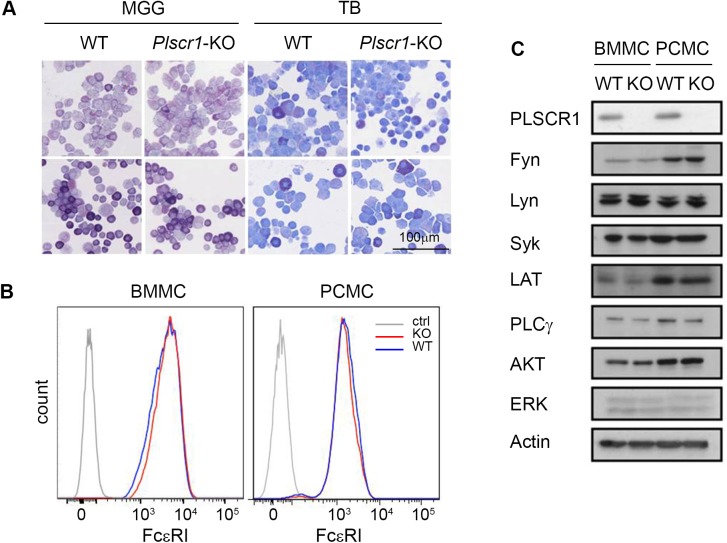
Phenotypic analysis of *Plscr1*^*-/-*^ BMMC and PCMC. (A) Cytologic analysis. Wild-type and *Plscr1*^*-/-*^ BMMC (top panels) and PCMC (bottom panels) were cyto-centrifuged and stained with May-Grünwald Giemsa (left, MGG) or toluidine blue (right, TB) and observed with an optical microscope. Scale bar = 100 μm. (B) The expression of FcεRI on the surface of WT (blue line) and *Plscr1*^*-/-*^ (red line) BMMC and PCMC was analyzed by flow cytometry. Gray line: isotype controls. Representative data of three independent experiments. (C) Expression of major effector molecules of FcεRI signaling. Lysates of WT and *Plscr1*^*-/-*^ (KO) BMMC and PCMC were analyzed by immunoblotting with specific antibodies for the presence of PLSCR1, Fyn, Lyn, Syk, LAT1, PLCγ1, AKT, Erk1/2 and actin.

Mast cells can exhibit different phenotypes depending on their microenvironment. Bone marrow-derived cultured mast cells (BMMCs) are related to mucosal mast cells with an immature phenotype, whereas peritoneal cell-derived cultured mast cells (PCMCs) are considered more mature and more related to mast cells present in connective tissue [33]. To examine whether PLSCR1 could affect differently mucosal-type and connective tissue-type mast cells, we generated BMMC and PCMC from *Plscr1*^*-/-*^ and *Plscr1*^*+/+*^ mice. The phenotype of these cells was first analyzed after staining with May-Grünwald Giemsa (MGG) and toluidine blue (TB). As seen in Fig 1A, PCMC had on average denser granules which were more heavily stained with MGG and TB. However no detectable difference could be observed between WT and *Plscr1*^*-/-*^ mast cells. As well, the expression of FcεRI on the surface of BMMC and PCMC showed no detectable difference between the WT and *Plscr1*^*-/-*^ cell populations (Fig 1B). The expression of major effectors of FcεRI signaling such as Fyn, Lyn, Syk, LAT1, Akt, PLCγ1 and Erk1/2 was identical between both genotypes whether in BMMC or PCMC (Fig 1C) despite a lower expression of Fyn, LAT1 and AKT in BMMC as compared to PCMC. Altogether, the phenotypic characterization of WT and *Plscr1*^*-/-*^ mast cells suggests that the absence of PLSCR1 does not affect significantly mast cell differentiation *in vitro*.

### PLSCR1 amplifies degranulation *in vitro* in primary cultures of mast cells

**Fig 2:**
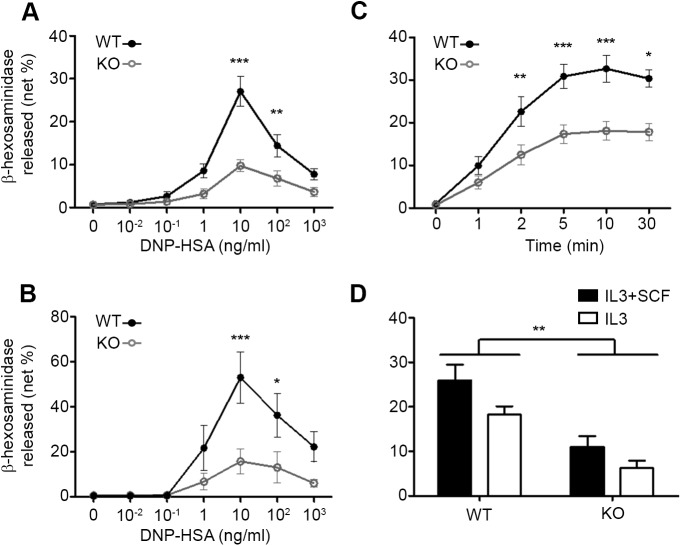
PLSCR1 amplifies degranulation *in vitro* in primary culture of BMMC and PCMC. Wild-type (WT) and *Plscr1*^*-/-*^ (KO) BMMC (A, C, D) and PCMC (B) were sensitized for 24 hours with anti-DNP IgE. After two washes, the IgE-sensitized cells were stimulated with different doses of specific antigen (DNP-HSA) for 30 minutes (A, B) or at different times to the optimal antigen dose of 10 ng/ml (C). Statistical analysis was done by a Two-way ANOVA followed by Sidak’s multiple comparisons test. Data of n independent experiments with n = 12 (A), n = 5 (B) and n = 6 (C) are presented as mean ± s.e.m. *: P <0.05; **: P <0.01; ***: P <0.001. (D) WT and KO BMMC generated in the presence of IL3 and SCF (black bars) or of IL3 alone (open bars) were stimulated for 30 min with 10 ng/ml DNP-HSA. Data of n independent experiments with n = 7 (IL3 + SCF) and n = 3 (IL3) are presented as mean ± s.e.m. Statistical analysis was done by an unpaired Student *t* test. **: p < 0.01.

To determine whether the absence of PLSCR1 could affect FcεRI-dependent degranulation of primary mast cells, we performed antigen dose-responses and time-courses. In the absence of PLSCR1, the IgE-dependent degranulation of BMMC (Fig 2A) and PCMC (Fig 2B) was reduced by more than 50% in dose-response experiments. This difference was not due to different degranulation kinetics between both genotypes since FcεRI-dependent degranulation of *Plscr1*^*+/+*^ and *Plscr1*^*-/-*^ BMMC reached a plateau 5 minutes after stimulation with no detectable difference in kinetics (Fig 2C). It has been shown that PLSCR1 may be involved in the response to SCF [8] and SCF is known to amplify FcεRI-dependent mast cell degranulation [3]. To determine whether the observed consequence of the absence of PLSCR1 was due to an effect on SCF-mediated signaling rather than to an effect on FcεRI signaling *per se*, we generated BMMC in the presence of IL3 with or without SCF. Although BMMC grown in the presence of SCF degranulated more extensively upon FcεRI engagement than BMMC derived without SCF, the absence of PLSCR1 affected BMMC in both cell culture conditions to a comparable extent (Fig 2D). Therefore our data demonstrate that the amplifier function of PLSCR1 previously observed in tumoral mast cells [6] is also operative in primary mast cells. They also extend this function previously observed in rat mast cells to mouse mast cells allowing to hypothesize that the amplifier function of PLSCR1 is not restricted to a particular species.

### PLSCR1 amplifies mast cell degranulation in an *in vivo* model of passive systemic anaphylaxis (PSA)

**Fig 3:**
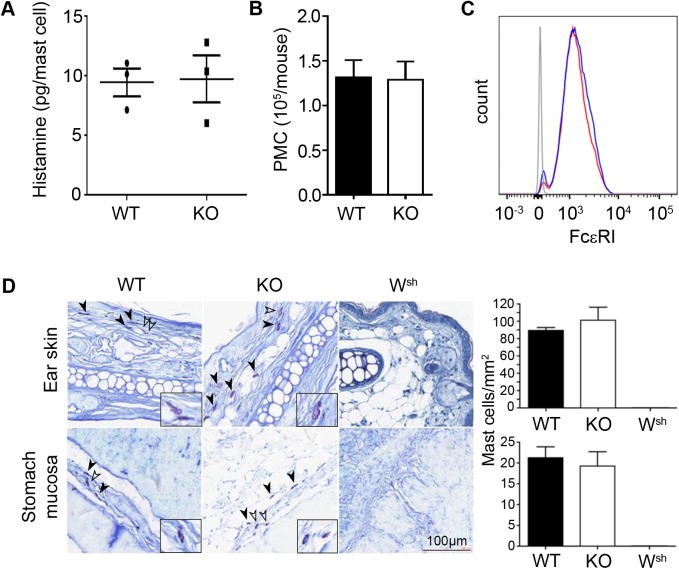
*Ex vivo* and *in vivo* analysis of *Plscr1*^*-/-*^ mast cells. (A) Histamine content of peritoneal mast cells from WT and *Plscr1*^*-/-*^ (KO) mice was measured by EIA and expressed as pg per mast cell. Values obtained for each individual mouse are shown. Data are presented as mean ± s.e.m.. Statistical analysis was done by an unpaired Student *t* test and showed no significant difference. (B) Number of peritoneal mast cells (PMC). The peritoneal cavity of WT and *Plscr1*^*-/-*^ (KO) mice was washed and the number of PMC per mouse was counted after staining with toluidine blue. Data are presented as mean ± s.e.m.. Statistical analysis done by an unpaired Student *t* test showed no significant difference. (C) The surface expression of FcεRIα chain by WT and *Plscr1*^*-/-*^ peritoneal mast cells was analyzed by flow cytometry. Blue line: WT, red line: *Plscr1*^*-/-*^, gray line: isotype controls. Representative data of three independent experiments. (D) Number and tissue localization of *Plscr1*^*-/-*^ mast cells. Left panels: Representative histological analysis of the ear skin and of the stomach submucosa of wild-type (WT), *Plscr1*^*-/-*^ (KO) and Kit^*W-sh/W-sh*^ (W^sh^) mice with toluidine blue staining. Arrowheads: mast cells, stained in purple. Selected mast cells (empty arrowheads) are shown under higher magnification in the insets. Scale bar: 100μm. Right panels: Quantitative analysis of countable mast cells in these tissues using CaloPix software. Data are presented as mean + s.e.m. of four mice. Statistical analysis done by an unpaired Student *t* test showed no significant difference.

To determine if the degranulation defect of *Plscr1*^*-/-*^ mast cells observed *in vitro* could have consequences *in vivo*, we first characterized *ex vivo* the mast cells of *Plscr1*^*-/-*^ mice. Peritoneal mast cells collected by lavage of the peritoneal cavity showed equivalent histamine content between *Plscr1*^*+/+*^ and *Plscr1*^*-/-*^ mice (Fig 3A) and their count was equivalent in both groups (Fig 3B). As well FcεRI expression was similar in both genotypes (Fig 3C). Histological analysis for the presence of connective tissue-type mast cells in the ear skin and of the mast cells present in the submucosa of stomach revealed that *Plscr1*^*-/-*^ mice had no detectable mast cell deficiency (Fig 3D).

Several established *in vivo* models are used to study specifically the reactivity of mast cells. We chose to use the passive systemic anaphylaxis (PSA) model which consists in an i.v. injection of IgE followed 24 hours after by an i.v. injection of the antigen recognized by this IgE. The anaphylactic response is then measured as a drop in body temperature due to the histamine released in the bloodstream during mast cell activation [34, 35].

**Fig 4:**
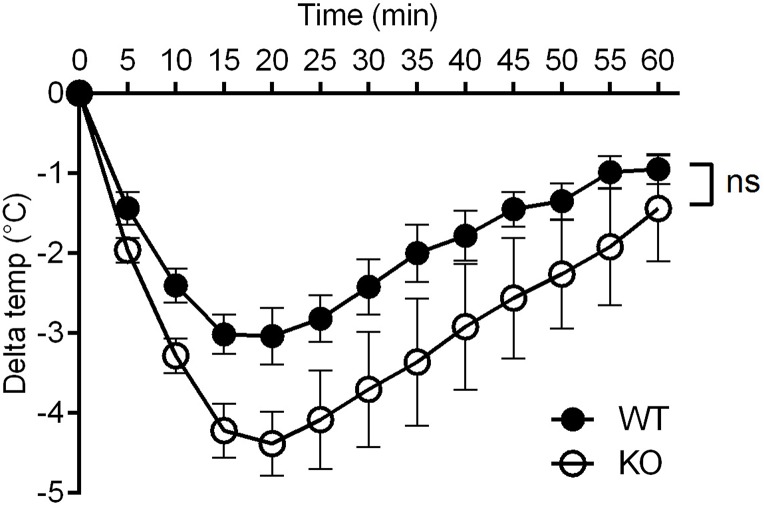
Sensitivity of *Plscr1*^*-/-*^ mice to histamine. Thermal probes were inserted under the dorsal skin of wild-type (WT) and *Plscr1*^*-/-*^ (KO) mice. The following day mice were injected i.v. with 5 μmol histamine. The drop in their body temperature was monitored using a thermal probe reader. Data are presented as ± s.e.m. with n = 6 mice per group (3 experiments). Statistical analysis was done using a two-way ANOVA. ns: non significant.

First, to rule out any effect due to a decreased response of tissues to histamine in *Plscr1*^*-/-*^ mice, we compared the drop in body temperature following direct injection of histamine in both genotypes. The two curves were not significantly different although there was a seemingly (but non significant) trend for an increased reactivity in *Plscr1*^*-/-*^ mice, suggesting rather a protective effect of PLSCR1 in tissue reactivity to histamine (Fig 4).

**Fig 5:**
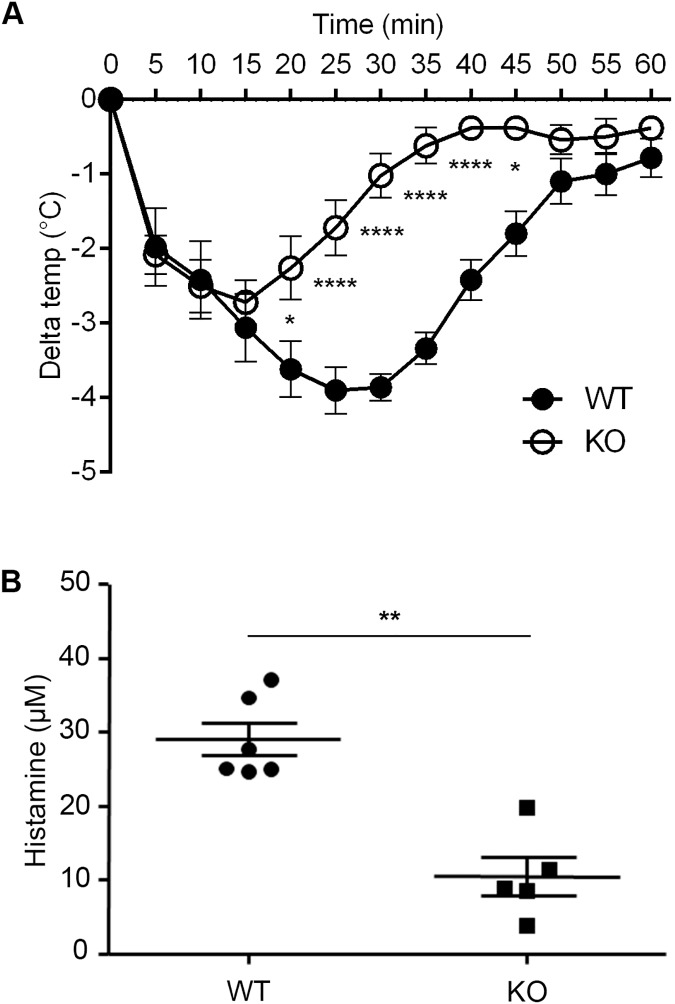
PLSCR1 amplifies anaphylactic reaction through increased mast cell degranulation. A passive systemic anaphylaxis (PSA) was carried out on wild-type (WT) and *Plscr1*^*-/-*^ (KO) mice. (A) PSA was monitored by following the drop in body temperature every five minutes after antigen injection (t = 0) using a thermal probe reader. Data are presented as mean ± s.e.m. with 5 mice (3 experiments). Two-way ANOVA followed by Sidak’s multiple comparisons test was used to compare the two groups. *: P < 0.05; ****: P < 0.0001. (B) PSA was monitored by measurement of the histamine released in the serum 25 minutes after antigen injection in the two groups of mice. Histamine was assayed using an EIA kit. The values obtained for each individual mouse are shown. Data are presented as mean ± s.e.m. Statistical analysis: unpaired Student *t* test. **: P <0.01.

We then performed PSA experiments. The *Plscr1*^*-/-*^ mice were protected from the reaction with a significantly reduced drop in body temperature compared to wild-type mice favoring a more rapid recovery of the former (Fig 5A). In agreement with the *in vitro* reduction in *Plscr1*^-/-^ mast cell degranulation (Fig 2), this reduced PSA reaction was associated with a decreased content of histamine released in the blood of these mice (Fig 5B).

Altogether, our data demonstrate that PLSCR1 amplifies FcεRI-dependent mouse mast cell degranulation *in vivo* thereby worsening anaphylactic reactions.

## Discussion

Mast cells express a large panel of receptors, including the FcεRI which binds IgE with a high affinity in an extremely stable manner, lasting for several weeks *in vivo* [36]. Mast cells are widely distributed in tissues where they can reside for months since we previously demonstrated that after depletion of peritoneal mast cells the peritoneal cavity of mice was repopulated very slowly by mast cells, reaching half of its original mast cell count after six months [37], a clear indication of their very slow turnover. Following their activation through various receptors, and notably after cross-linking of their FcεRI, these cells are capable to release within a couple of minutes and in the long term a large array of extremely potent mediators. This endows mast cells with the ability to act as a particularly powerful very first line of defense against infectious agents and to neutralize/degrade toxins from venoms and environmental irritants [38, 39]. However, when control over this system is lost, its activation can lead to the development of a variety of diseases such as allergy. Identification of modulators of mast cell responses to allergenic stimulation for targeted therapeutic intervention is therefore an important task to restore controlled activation of these cells. We report here that PLSCR1 is one such modulator.

We have previously identified PLSCR1 as an amplifier of mast cell degranulation following FcεRI engagement in the rat tumoral mast cell line RBL-2H3 [6]. The relevance of this observation in primary mast cells and in *in vivo* anaphylactic reactions remained to be evaluated. In the present study, taking advantage of *Plscr1*^*-/-*^ mice, we demonstrate that PLSCR1 amplifies mast cell degranulation of variously mature non-tumoral mast cells. Thus in the absence of PLSCR1, the FcεRI-mediated mast cell degranulation was decreased at all antigen doses in BMMC (a model of mucosal-type immature mast cells) and in the more mature model of PCMC. In addition, histamine released in serum during PSA, a well-established model of IgE-dependent anaphylactic reaction in which mast cells play a central role [34, 40], was also significantly reduced in *Plscr1*^*-/-*^ mice demonstrating that PLSCR1 controls the extent of mast cell activation in their physiologic tissue environment. This was accompanied by a reduced anaphylactic reaction (i.e. reduced drop in body temperature) showing that PLSCR1 controls the severity of anaphylaxis. Yet, the anaphylactic reaction induced by direct injection of histamine was slightly increased in *Plscr1*^*-/-*^ animals, strongly indicating that PLSCR1 controls the IgE-dependent anaphylactic reaction through its control of mast cell activation (i.e. the extent of histamine released) rather than through a control of the sensitivity of tissues to histamine.

The number of studies addressing the role of PLSCR1 *in vivo* is limited. There is no detectable phenotype associated with PLSCR1 deficiency in adult mice in steady-state conditions [8]. However, a transient neutropenia in the newborn *Plscr1*^*-/-*^ mice was reported that was related to a defect in stress granulopoiesis ("emergency granulopoiesis") due to a decreased response to G-CSF [41]. Also, PLSCR1 is one of the proteins produced in response to type I interferon [21] and it was demonstrated that it protects mice from Staphylococcal α-toxin [42], a protein that promotes type I interferon responses. Here we report that, although PLSCR1 amplifies anaphylactic reactions, the phenotype of resting mast cells was not detectably altered by the absence of PLSCR1 whether in cell cultures or *ex vivo* in peritoneal mast cells. As well, the distribution of mast cells in the examined tissues was not impacted by the absence of PLSCR1 in adult mice. We conclude that PLSCR1 might not play a major role *in vivo* in steady-state conditions but that it plays an important role *in vivo* when homeostasis is broken. Thus our current hypothesis is that one of the main function of PLSCR1 is to regulate activation signals. Supporting this hypothesis, PLSCR1 was previously reported to amplify responses to receptors for EGF [27], SCF [8], and G-CSF [41], to TLR9 [43] and apoptotic stimuli [12]. By contrast, PLSCR1 can dampen IgG-dependent phagocytosis [44]. Thus a general picture emerges in which, dependent on the context, PLSCR1 can intervene to either increase or decrease biological responses to disrupted homeostasis. We believe that this modulating ability is a major function of this protein.

The mechanism allowing PLSCR1 to fulfill amplification of FcεRI signaling remains to be deciphered. In the RBL-2H3 model, tyrosine phosphorylation of the adapter protein LAT and of phospholipase C-γ1 (PLC-γ1) as well as calcium mobilization are strongly reduced in the virtual absence of PLSCR1 [6]. However, no effect on the phosphorylation of Akt and ERK was observed, indicating that PLSCR1 is a selective amplifier of the signaling axis initiated by Lyn and involving LAT, PLC-γ1 and calcium influx, i.e. one of the major pathways leading to mast cell degranulation following FcεRI-dependent cell activation. PLSCR1 is also in part constitutively localized in lipid rafts and associated with Lyn and Syk but not LAT or Fyn [6]. However, it does not appear to modulate Lyn activity, suggesting that PLSCR1 intervenes downstream of Lyn. Since PLSCR1 tyrosine phosphorylation is also dependent on Lyn, Syk and calcium mobilization (i.e. on the same signaling axis) after FcεRI engagement [28, 29], the relationship between its phosphorylation on tyrosine residues and its function could shed light on its mechanism of action. This will be explored in future studies.

Finally, the fact that PLSCR1 aggravates the severity of anaphylactic reactions in a murine model raises the possibility that perturbation of its function (e.g. increased amplification mechanism or loss of proper control) might play a role in some allergic patients. Studies in human mast cells are now required to address this question.

In conclusion, we report for the first time the physiological relevance of PLSCR1 in the severity of anaphylactic reactions *in vivo* through its control of IgE-dependent mast cell degranulation.
